# International travel between global urban centres vulnerable to yellow fever transmission

**DOI:** 10.2471/BLT.17.205658

**Published:** 2018-04-11

**Authors:** Shannon E Brent, Alexander Watts, Martin Cetron, Matthew German, Moritz UG Kraemer, Isaac I Bogoch, Oliver J Brady, Simon I Hay, Maria I Creatore, Kamran Khan

**Affiliations:** a Li Ka Shing Knowledge Institute, St. Michael's Hospital, 30 Bond Street, Toronto, Ontario, M5B 1W8, Canada.; b Division of Global Migration and Quarantine, National Center for Emerging and Zoonotic Infectious Diseases, Atlanta, United States of America (USA).; c Computational Epidemiology Laboratory, Boston Children’s Hospital, Boston, USA.; d Divisions of General Internal Medicine and Infectious Diseases, University Health Network, Toronto, Canada.; e Centre for the Mathematical Modelling of Infectious Diseases, London School of Hygiene and Tropical Medicine, London, England.; f Institute for Health Metrics and Evaluation, University of Washington, Seattle, USA.; g Dalla Lana School of Public Health, University of Toronto, Toronto, Canada.

## Abstract

**Objective:**

To examine the potential for international travel to spread yellow fever virus to cities around the world.

**Methods:**

We obtained data on the international flight itineraries of travellers who departed yellow fever-endemic areas of the world in 2016 for cities either where yellow fever was endemic or which were suitable for viral transmission. Using a global ecological model of dengue virus transmission, we predicted the suitability of cities in non-endemic areas for yellow fever transmission. We obtained information on national entry requirements for yellow fever vaccination at travellers’ destination cities.

**Findings:**

In 2016, 45.2 million international air travellers departed from yellow fever-endemic areas of the world. Of 11.7 million travellers with destinations in 472 cities where yellow fever was not endemic but which were suitable for virus transmission, 7.7 million (65.7%) were not required to provide proof of vaccination upon arrival. Brazil, China, India, Mexico, Peru and the United States of America had the highest volumes of travellers arriving from yellow fever-endemic areas and the largest populations living in cities suitable for yellow fever transmission.

**Conclusion:**

Each year millions of travellers depart from yellow fever-endemic areas of the world for cities in non-endemic areas that appear suitable for viral transmission without having to provide proof of vaccination. Rapid global changes in human mobility and urbanization make it vital for countries to re-examine their vaccination policies and practices to prevent urban yellow fever epidemics.

## Résumé

**Objectif:**

Évaluer la capacité des déplacements internationaux à propager le virus de la fièvre jaune dans des villes du monde entier.

**Méthodes:**

Nous avons recueilli des données sur les itinéraires de vols internationaux de voyageurs qui quittaient des zones d'endémie de la fièvre jaune à travers le monde en 2016 pour se rendre dans des villes où la fièvre jaune était endémique ou qui étaient propices à la transmission virale. À l'aide d'un modèle écologique mondial de transmission du virus de la dengue, nous avons prédit le caractère approprié de villes situées dans des zones non endémiques pour la transmission de la fièvre jaune. Nous avons obtenu des informations sur les conditions nationales d'entrée relatives à la vaccination contre la fièvre jaune dans les villes d'arrivée des voyageurs.

**Résultats:**

En 2016, 45,2 millions de passagers de vols internationaux ont quitté des zones d'endémie de la fièvre jaune situées dans le monde entier. Sur 11,7 millions de voyageurs à destination de 472 villes où la fièvre jaune n'était pas endémique, mais qui étaient propices à la transmission du virus, 7,7 millions (65,7%) n'ont pas eu à fournir de preuve de vaccination à leur arrivée. C'est au Brésil, en Chine, aux États-Unis d'Amérique, en Inde, au Mexique et au Pérou que les volumes de voyageurs arrivant de zones d'endémie de la fièvre jaune étaient les plus élevés et que le nombre de personnes vivant dans des villes propices à la transmission de la fièvre jaune était le plus important.

**Conclusion:**

Chaque année, des millions de voyageurs quittent, sans avoir à fournir de preuve de vaccination, des zones d'endémie de la fièvre jaune à travers le monde pour se rendre dans des villes situées dans des zones non endémiques qui s'avèrent propices à la transmission virale. Compte tenu de l'évolution rapide de la mobilité humaine et de l'urbanisation dans le monde entier, il est indispensable que les pays réexaminent leurs politiques et pratiques de vaccination pour prévenir les épidémies de fièvre jaune en milieu urbain.****

## Resumen

**Objetivo:**

Examinar el potencial de los viajes internacionales de propagar el virus de la fiebre amarilla en ciudades de todo el mundo.

**Métodos:**

Se obtuvieron datos de los itinerarios de vuelo internacionales de viajeros que partieron de áreas endémicas de la fiebre amarilla en el mundo en 2016 hacia ciudades donde la fiebre amarilla era endémica o que eran adecuadas para la transmisión viral. Mediante el uso de un modelo ecológico global de la transmisión del virus del dengue, se predijo la idoneidad de las ciudades en áreas no endémicas para la transmisión de la fiebre amarilla. Se obtuvo información sobre los requisitos nacionales de entrada sobre la vacunación contra la fiebre amarilla en las ciudades de destino de los viajeros.

**Resultados:**

En 2016, 45,2 millones de viajeros de vuelos internacionales salieron de áreas endémicas de fiebre amarilla en el mundo. De los 11,7 millones de viajeros con destinos en 472 ciudades donde la fiebre amarilla no era endémica, pero eran aptas para la transmisión viral, 7,7 millones (65,7%) no estaban obligados a proporcionar una prueba de vacunación al llegar. Brasil, China, la India, México, Perú y los Estados Unidos tenían los mayores volúmenes de viajeros que provenían de áreas endémicas de fiebre amarilla y las mayores poblaciones en ciudades adecuadas para la transmisión de la fiebre amarilla.

**Conclusión:**

Cada año, millones de viajeros abandonan las áreas endémicas de la fiebre amarilla del mundo hacia ciudades de áreas no endémicas que parecen adecuadas para la transmisión viral sin tener que presentar una prueba de vacunación. Los rápidos cambios globales en la movilidad humana y la urbanización hacen que sea vital que los países reexaminen sus políticas y prácticas de vacunación para prevenir las epidemias urbanas de fiebre amarilla.

## ملخص

**الغرض:**

فحص احتمالية أن يؤدي السفر حول العالم إلى انتشار فيروس الحمّى الصفراء في مدن العالم.

**الطريقة:**

لقد حصلنا على مجموعة من البيانات بشأن قوائم الرحلات الدولية التي تضم المسافرين الذين غادروا من المناطق التي تعاني من الحمّى الصفراء حول العالم في عام 2016، متجهين إلى مدن كانت تعاني من انتشار الحمّى الصفراء بها أو التي كانت مواتية لانتشار العدوى الفيروسية بها. وباستخدام نموذج بيئي عالمي لانتقال فيروس الضنك، قمنا بالتنبؤ بمدى ملاءمة المدن التي تقع في مناطق غير مصابة بالحمّى لانتقال الحمّى الصفراء إليها. وحصلنا على معلومات عن المتطلبات الوطنية للسماح بإدخال المسافرين فيما يتعلق بتطعيم الحمّى الصفراء في المدن التي يتجه إليها المسافرون.

**النتائج:**

في عام 2016، غادر 45.2 مليون مسافر عبر الرحلات الجوية الدولية من مناطق تعاني من الحمّى الصفراء حول العالم. ومن بين 11.7 مليون مسافر اتجهوا إلى 472 مدينة لم تعاني من انتشار الحمّى الصفراء بها لكنها كانت ملائمة لانتقال الفيروس بها، لم يكن 7.7 مليون من المسافرين (بنسبة 65.7‏%) مُطالبين بتقديم إثبات حول حصولهم على التطعيم عند الوصول. وقد تلقت البرازيل والصين والهند والمكسيك وبيرو والولايات المتحدة الأمريكية الأعداد الأكبر من المسافرين الذين وصلوا من مناطق مصابة بالحمّى الصفراء وكانوا يمثلون نسبة السكان الأكبر الذي يعيشون في مدن ملائمة لانتقال الحمّى الصفراء إليها.

**الاستنتاج:**

يغادر الملايين من المسافرين في كل عام من مناطق مصابة بالحمّى الصفراء حول العالم إلى مدن تقع في مناطق غير مصابة بالعدوى والتي تبدو أنها ملائمة لانتقال الفيروس إليها دون الحاجة إلى تقديم إثبات على الحصول على التطعيم. إن التغييرات العالمية السريعة في حركة البشر وتكوين المجتمعات الحضرية تحتم على البلدان إعادة النظر في سياسات وممارسات توفير التطعيمات لديها لتفادي حدوث عدوى الحمّى الصفراء. ****

## 摘要

**目的:**

审核黄热病病毒通过国际旅行在全球各城市传播的可能性。

**方法:**

我们获取了相关旅客国际航班线路的行程信息。这些旅客于 2016 年从全球黄热病流行区出发前往各个城市。这些目的地城市要么是黄热病流行区，要么是病毒易感区。我们运用登革热病毒传播的全球生态模型预测非病区城市的黄热病易感性。我们获取了旅客抵达的目的地城市针对黄热病疫苗接种的入境要求信息。

**结果:**

2016 年，4520 万搭乘国际航班的旅客从全球黄热病流行区出发。其中，1170 万旅客的目的地位于 472 个非黄热病流行区的城市，但这些城市属于病毒易感区，770 万 (65.7%) 的旅客在抵达时并未被要求提供疫苗接种证明。巴西、秘鲁、墨西哥、美国、印度和中国拥有最多来自黄热病流行区的旅客，同时这些国家城市人口最为密集，是黄热病病毒传播的易感区。

**结论:**

每年都有数百万旅客从全球黄热病流行区离开并前往非病区但易于传播病毒的城市，并且无需提供疫苗接种证明。随着全球人口移动性的增加和城市化进程的加快，于各国而言，重新审视其疫苗接种政策和惯例以预防黄热病爆发至关重要****

## Резюме

**Цель:**

Изучить потенциальное влияние международных поездок на распространение вируса желтой лихорадки в городах по всему миру.

**Методы:**

Авторы получили данные о маршрутах международных перелетов путешественников, которые в 2016 году отправлялись из районов мира, эндемичных по желтой лихорадке, для тех городов, где желтая лихорадка не была эндемичной или в которых были благоприятные условия для передачи вируса. Используя глобальную экологическую модель передачи вируса денге, авторы предсказали наличие благоприятных условий для передачи желтой лихорадки в городах в неэндемичных районах. Авторы получили информацию о национальных требованиях на въезд для вакцинации против желтой лихорадки в городах назначения путешественников.

**Результаты:**

В 2016 году 45,2 миллиона пассажиров международных авиарейсов совершили вылеты из районов мира, эндемичных по желтой лихорадке. Из 11,7 миллиона путешественников с пунктами назначения в 472 городах, где желтая лихорадка не была эндемичной, но в которых имелись благоприятные условия для передачи вируса, 7,7 миллиона (65,7%) не были обязаны по прибытии предоставлять подтверждающие документы о вакцинации. В Бразилии, Индии, Китае, Мексике, Перу и Соединенных Штатах Америки было наибольшее число путешественников, прибывающих из районов, эндемичных по желтой лихорадке. А также в этих странах наибольшее число людей проживает в городах с благоприятными условиями для передачи вируса желтой лихорадки.

**Вывод:**

Каждый год миллионы путешественников отправляются из районов мира, эндемичных по желтой лихорадке, в города в неэндемичных районах, которые, по-видимому, пригодны для передачи вируса, без необходимости предоставления подтверждающих документов о вакцинации. Стремительные глобальные изменения в мобильности людей и урбанизации делают для стран жизненно важным вопрос пересмотра своей политики и практики вакцинации для предотвращения эпидемий желтой лихорадки в городах.

## Introduction

In December 2015, Angola reported its first locally acquired case of yellow fever in nearly a decade. The ensuing epidemic was first recognized in Luanda, then spread across Angola’s 18 provinces, resulting in 4347 suspected or confirmed cases and 377 deaths.^1^ International travellers departing from Angola then imported yellow fever virus into Kenya and the Democratic Republic of the Congo,^2^ where another epidemic ensued, causing 2987 suspected or confirmed cases and 121 deaths.^1^ Furthermore, 11 foreign workers infected in Angola travelled to urban centres in China, the first time imported cases of yellow fever have been reported in Asia.^3^ Four cases were recently imported into Europe over an 8-month period by travellers returning from South America.^4^ The time period is in stark contrast to the 27 years during which the previous four cases of travel-associated yellow fever were imported into Europe.^4^ In early 2018, nine cases were exported from Brazil and led to three deaths.^5^ Increased air travel and globalization is making it easier for humans to transport yellow fever virus across international borders, potentially catalysing deadly urban epidemics.^3^

An essential tool in the fight against yellow fever is a live-attenuated vaccine developed in 1937.^6^ This vaccine is vital for the prevention and control of yellow fever epidemics since no effective antiviral therapy exists.^7^ However, a substantial proportion of the world’s yellow fever vaccine stock was recently consumed in response to epidemics in Africa^8^ and Brazil.^9^ As a stopgap measure, the World Health Organization (WHO) approved fractional dosing to extend the vaccine supply, while recognizing that the duration of immunity may be compromised.^10^ With only four WHO-qualified yellow fever vaccine manufacturers in the world, rapid replenishment of the global emergency stockpile stretches finite resources, potentially resulting in vaccine shortages for preventive campaigns.^11^ In late 2017, stocks of YF-VAX^®^ (Sanofi Pasteur, Lyon, France) in North America were depleted because of manufacturing difficulties.^5^ Should another urban epidemic occur in the near future, vaccine demand could easily exceed the available supply.

Although many countries have vaccination policies to prevent international spread of the yellow fever virus, implementation is inconsistent.^12^ Most, but not all countries where yellow fever is endemic require arriving international travellers without medical contraindications to provide official documentation of vaccination as a prerequisite for entry. As the vaccine provides protective immunity to 90% and 99% of individuals 10 and 30 days after vaccination, respectively,^13^ most travellers are protected from acquiring and exporting the yellow fever virus. Furthermore, some countries where the disease is not endemic, but where the competent mosquito vector *Aedes aegypti* is present require travellers arriving from a yellow fever-endemic country to provide proof of vaccination.^14^

The confluence of climate change,^15^ rapid urbanization^16^ and international air travel^17^ are accelerating the globalization of mosquito-borne viruses such as dengue, chikungunya and Zika viruses. Here we examined the potential for the yellow fever virus to spread via international air travel into the world’s cities, in order to guide global epidemic prevention efforts.

## Methods

To identify gaps in yellow fever vaccination policies around the world, we assessed the potential for the international spread of yellow fever from areas deemed by WHO to be at risk of transmission to areas where conditions are known, or predicted, to be suitable for transmission. Our goal was to provide a global perspective on urban exposure to imported yellow fever virus, irrespective of past or present epidemics.

### Global endemicity

We considered places where WHO recommended yellow fever vaccination in 2016, including recently identified parts of Brazil, to be areas where humans were at risk of local infection.^18^^–^^20^ We refer to these areas as yellow fever-endemic areas, although we recognize that they may not have been experiencing yellow fever transmission. We excluded places where yellow fever vaccination was generally not recommended by WHO. For non-holoendemic countries (i.e. where only part of the country was at risk of yellow fever),^20^ we delineated subnational areas of risk using ArcGIS v. 10.4.1 (Esri, Redlands, United States of America). We then used LandScan (Oak Ridge National Laboratory, Oak Ridge, USA)^21^ to estimate the total population living within the global range of the yellow fever virus.

### International dispersion

To account for the possibility that individuals infected with yellow fever virus within an endemic area might travel by land to a nearby airport in a non-endemic area, we used ArcGIS v. 10.4.1 to identify all commercial airports registered with the International Air Transport Association (IATA): (i) within 200 km of any yellow fever-endemic area worldwide (base scenario); and (ii) within 200 km of any city within a yellow fever-endemic area (urban scenario). In the base scenario, we considered travellers departing from areas of potential sylvatic or urban transmission as possible sources of exported yellow fever virus. In the urban scenario, we focused on travellers departing from airports within 200 km of a city (i.e. an urban centre with more than 300 000 residents, as defined by the United Nations’ World Urbanization Prospects)^22^ located in a yellow fever-endemic area. We mapped the final destination airports and the number of international travellers (determined from unique trips on commercial flights) departing from airports in each scenario by analysing worldwide tickets sales data from IATA between 1 January and 31 December 2016.^23^ These data included the travellers’ full itineraries: their initial airport of embarkation, their final destination airport and, where applicable, connecting airports. The data did not detail uncompleted trips due, for example, to cancelled or missed flights. Overall, these data accounted for an estimated 90% of all trips on commercial flights worldwide; the remaining 10% were modelled using airline market intelligence.^23^ Such data have been used previously to anticipate the global spread of emerging infectious diseases.^24^

### Potential for urban transmission

To identify cities where yellow fever was not endemic, but which may have been suitable for viral transmission, we used a high-resolution, global, ecological model of dengue virus transmission, which was developed using empirical data on the real-world occurrence of dengue fever and associated environmental and climatic predictors of dengue virus transmission.^25^ We assumed that cities predicted to be suitable for dengue virus transmission were also ecologically suitable for yellow fever virus transmission, because both viruses are primarily transmitted by *Aedes aegypti*, an anthropophilic mosquito highly adapted to urban settings.^25^ Adopting a conservative approach, we excluded cities where the predicted probability of dengue-suitability was below 50%. As our analysis focused on urban importation and transmission of yellow fever virus, we did not consider its introduction into rural, sylvatic areas or transmission among non-human primates. We defined a yellow fever-suitable city as a population centre with at least 300 000 residents in an area where the yellow fever virus was not endemic but which was predicted to be suitable for viral transmission. We excluded cities above 2300 m because environmental conditions at these elevations are considered unsuitable for yellow fever virus transmission.^26^

We assessed the potential for importation of the yellow fever virus by quantifying the volume of airline passengers travelling from yellow fever-endemic areas of the world, according to our base and urban scenarios, to yellow fever-suitable and -endemic cities. We also considered the possibility that individuals infected with the virus might arrive at an airport in a non-endemic area and then travel by land to a neighbouring city within a yellow fever-endemic or -suitable area: in our analysis, we included all commercial airports located within 200 km of these mutually exclusive geographical areas. We then categorized traveller flows according to the official yellow fever travel vaccination policy in each endemic and non-endemic country: (i) no proof of yellow fever vaccination required; (ii) proof of vaccination required if arriving from a yellow fever-endemic country; and (iii) proof of vaccination required if arriving from any country.^27^ Finally, we aggregated the resident populations of all yellow fever-suitable and -endemic cities.

## Results

We estimated that 923 million people lived in areas of the world where yellow fever was endemic in 2016, spanning 25 holoendemic and 17 non-holoendemic countries or territories (Box 1).

Box 1 **Countries and territories at risk of yellow fever transmission in 2016, according to the United States’ Centers for Disease Control and Prevention and the World Health Organization^1^^–^^3^**

***Countries and territories where yellow fever was endemic (i.e. holoendemic countries)***

Angola, Benin, Burkina Faso, Burundi, Cameroon, Central African Republic, Congo, Côte d'Ivoire, Equatorial Guinea, French Guiana, Gabon, Gambia, Ghana, Guinea, Guinea-Bissau, Guyana, Liberia, Nigeria, Paraguay, Senegal, Sierra Leone, South Sudan, Suriname, Togo, Uganda

***Countries where only a portion were at risk of yellow fever (i.e. non-holoendemic countries)***

Argentina, Bolivia (Plurinational State of), Brazil, Chad, Colombia, Democratic Republic of the Congo, Ecuador, Ethiopia, Kenya, Mali, Mauritania, Niger, Panama, Peru, Sudan, Trinidad and Tobago, Venezuela (Bolivarian Republic of)

**Fig. 1:**
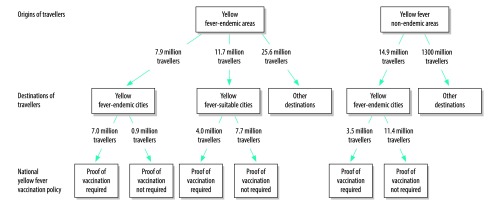
International movements of air travellers between areas that were or were not endemic for yellow fever, 2016

Notes: A yellow fever-endemic area was a national or subnational area where the World Health Organization recommended yellow fever vaccination. A yellow fever-endemic city was a city located in an area where vaccination was recommended. A yellow fever-suitable city was a city that was suitable for dengue virus transmission (see main text for details). Other destinations were: (i) all destinations where yellow fever was not endemic and which were not suitable for yellow fever transmission; and (ii) areas where yellow fever was endemic or which were suitable for yellow fever transmission but did not contain a settlement with a population greater than 300 000.

In our base scenario, 45.2 million travellers departed from yellow fever-endemic areas for international destinations in 2016. Of these, 7.9 million (17.4%) had final destinations at airports within or adjacent to yellow fever-endemic cities, 11.7 million (25.8%) had destinations at airports within or adjacent to yellow fever-suitable cities and 25.6 million (57.8%) had other destinations (Fig. 1). Of the 7.9 million travellers with international destinations at or near other yellow fever-endemic cities, 0.86 million (11.0%) landed in a country where proof of yellow fever vaccination was not required upon arrival: one holoendemic country (i.e. South Sudan) and three non-holoendemic countries (i.e. Argentina, Brazil and Peru). Of the 11.7 million travellers with destinations at or near yellow fever-suitable cities, 7.7 million (65.7%) landed in a country where proof of yellow fever vaccination was not required: four non-holoendemic countries (i.e. Argentina, Brazil, Ecuador and Peru) and 12 non-endemic countries (e.g. the United States). Conversely, 14.9 million travellers departed non-endemic areas of the world for airports within or adjacent to yellow fever-endemic cities; 11.4 million (76.4%) of these travellers landed in countries where proof of yellow fever vaccination was not required on arrival.

In our urban scenario, 32.2 million travellers departed airports within or near yellow fever-endemic cities for international destinations in 2016. Of these, 6.1 million (18.9%) arrived at or near yellow fever-endemic cities (Table 1); there was one fewer destination city than in our base scenario. In addition, 8.4 million (26.1%) arrived at or near yellow fever-suitable cities; there were six fewer destination cities than in our base scenario (Table 2). As the urban scenario considered only travellers departing from airports within 200 km of a city within a yellow fever-endemic area, it represents the potential for dispersion during an urban outbreak rather than dispersion secondary to urban or sylvatic transmission, as in the base scenario.

**Table 1:** International air travellers arriving in cities where yellow fever was endemic from other endemic areas or cities, 2016

Destination country or territory,^a^ by rank^b^	No. travellers arriving from yellow fever-endemic areas		Urban population of destination country, millions^c^	Proof of yellow fever vaccination required upon arrival	
	Departure airport within 200 km of a yellow fever-endemic area (base scenario)^d^	Departure airport within 200 km of a city in a yellow fever-endemic area (urban scenario)^e^		From yellow fever-endemic countries only	From any country
1. Colombia	1 373 439	776 317	16.4	Yes	No
2. Panama	995 941	625 764	1.7	Yes	No
3. Brazil	769 203	474 260	54.6	No^f^	No^f^
4. Nigeria	532 602	485 319	46.8	Yes	No
5. Ghana	389 242	378 893	6.1	No	Yes
6. Côte d'Ivoire	360 179	347 372	6.0	No	Yes
7. Kenya	357 561	291 022	5.7	Yes	No
8. Senegal	322 374	295 805	3.5	Yes	No
9. Cameroon	280 895	272 308	7.5	Yes	No
10. Venezuela (Bolivarian Republic of)	221 837	185 895	7.3	Yes	No
11. Gabon	199 560	197 595	0.7	No	Yes
12. Congo	195 571	178 963	2.9	No	Yes
13. Benin	189 191	186 575	1.4	Yes	No
14. Mali	161 064	151 877	2.5	No	Yes
15. Paraguay	151 425	112 640	2.8	Yes	No
16. Uganda	149 683	135 482	1.9	Yes	No
17. Angola	125 518	92 021	7.2	No	Yes
18. Bolivia (Plurinational State of)	121 798	93 353	2.1	Yes	No
19. Democratic Republic of the Congo	118 798	80 433	20.1	No	Yes
20. Burkina Faso	105 837	97 019	3.5	Yes	No
21. Togo	104 851	102 487	1.0	No	Yes
22. South Sudan	92 280	83 838	0.3	No	No
23. Sudan	90 271	48 908	2.1	Yes	No
24. Guinea	75 603	73 078	1.9	Yes	No
25. Liberia	65 060	64 915	1.3	No	Yes
Other countries^g^	315 213	284 692	7.4	NA	NA
**Total**	**7 864 996**	**6 116 831**	**214.7**	**NA**	**NA**

NA: not applicable.

^a^ All destination countries and territories were yellow fever-endemic areas.

^b^ Countries and territories were ranked according to the number of travellers arriving from yellow fever-endemic areas, which was determined by examining all outbound international flights from airports within areas where the World Health Organization (WHO) recommended yellow fever vaccination and all airports within 200 km of such areas.^17^^–^^19^

^c^ Nationally aggregated population living in cities.

^d^ The base scenario considered international travellers arriving from airports within areas where WHO recommended yellow fever vaccination and all airports within 200 km of such areas.

^e^ The urban scenario considered international travellers arriving from airports within 200 km of a city (population ≥ 300 000) in an area where WHO recommended yellow fever vaccination.

^f^ We did not take into account Brazil’s temporary yellow fever vaccination requirements for incoming passengers from Angola and the Democratic Republic of the Congo during the 2016 outbreak.

^g^ There were 10 other yellow fever-endemic destination countries with an airport within 200 km of a yellow fever-endemic city with a population of at least 300 000: Argentina, Burundi, Central African Republic, Chad, Ethiopia, Gambia, Guinea-Bissau, Niger, Peru and Sierra Leone. We did not show the 7 countries where there was no city with at least 300 000 residents located in a yellow fever-endemic area: Ecuador, Equatorial Guinea, French Guiana, Guyana, Mauritania, Suriname and Trinidad and Tobago.

**Table 2:** International air travellers arriving in cities suitable for yellow fever transmission from areas or cities where yellow fever was endemic, 2016

Destination country or territory,^a^ by rank^b^	No. travellers arriving from yellow fever-endemic areas		Urban population of destination country, millions^c^	Proof of yellow fever vaccination required upon arrival	
	Departure airport within 200 km of a yellow fever-endemic area (base scenario)^d^	Departure airport within 200 km of a city in a yellow fever-endemic area (urban scenario)^e^		From yellow fever-endemic countries only	From any country
1. United States^f^	2 762 081	1 659 163	9.6	No	No
2. Mexico	1 166 021	874 820	33.5	No	No
3. United Arab Emirates	890 623	717 232	0.5	No	No
4. Peru	752 113	536 161	12.1	No	No
5. Ecuador	595 181	405 106	3.0	No	No
6. Dominican Republic	538 042	322 848	3.5	No	No
7. Brazil	481 737	311 969	44.2	No^g^	No^g^
8. Venezuela (Bolivarian Republic of)	461 006	376 804	7.6	Yes	No
9. China	403 683	316 588	98.7	Yes	No
10. India	385 786	345 314	235.3	Yes	No
11. Cuba	372 455	237 228	3.2	Yes	No
12. Saudi Arabia	319 711	256 316	6.5	Yes	No
13. Costa Rica	283 169	216 087	1.2	Yes	No
14. United Republic of Tanzania	268 038	247 515	7.8	Yes	No
15. Egypt	217 597	204 251	22.8	Yes	No
16. Argentina	213 665	170 456	6.3	No	No
17. Rwanda	170 040	162 831	1.3	Yes	No
18. Guatemala	115 834	94 882	2.9	Yes	No
19. El Salvador	103 943	85 577	1.1	Yes	No
20. China, Hong Kong SAR	96 258	74 284	7.3	No	No
21. Sudan	90 037	48 723	5.6	Yes	No
22. Thailand	86 481	62 266	12.7	Yes	No
23. Puerto Rico	77 282	57 657	2.8	No	No
24. Jamaica	76 848	19 822	0.6	Yes	No
25. Nicaragua	68 481	59 128	1.0	No	No
Other countries^h^	665 455	531 709	211.0	NA	NA
**Total**	**11 661 567**	**8 394 737**	**742.1**	**NA**	**NA**

NA: not applicable; SAR: Special Administrative Region.

^a^ Destination cities in these countries and territories were ecologically suitable for yellow fever virus transmission but were not in yellow fever-endemic areas.

^b^ Countries and territories were ranked according to the number of travellers arriving from yellow fever-endemic areas, which was determined by examining all outbound international flights from airports within areas where the World Health Organization (WHO) recommended yellow fever vaccination and all airports within 200 km of such areas.^17^^–^^19^

^c^ Nationally aggregated population living in yellow fever-suitable cities. In the urban scenario, there were six fewer yellow fever-suitable destination cities than in the base scenario: Satna, India (population 0.31 million); Ibb, Yemen (population 0.45 million); Al Hudaydah, Yemen (population 0.57 million); Taiz, Yemen (population 0.69 million); Aden, Yemen (population 0.88 million); and Sana’a, Yemen (population 2.7 million).

^d^ Our base scenario considered international travellers arriving from airports within areas where WHO recommended yellow fever vaccination and all airports within 200 km of such areas.

^e^ Our urban scenario considered international travellers arriving from airports within 200 km of a city (population ≥ 300 000) in an area where WHO recommended yellow fever vaccination.

^f^ United States’ territory included all continental states and Hawaii. Puerto Rico was not included and is listed separately. Other United States territories, such as Guam, American Samoa and the United States Virgin Islands, do not have cities with at least 300 000 residents and are thus not included.

^g^ We did not take into account Brazil’s temporary yellow fever vaccination requirements for incoming passengers from Angola and the Democratic Republic of the Congo during the 2016 outbreak.

^h^ There were 29 other countries or territories suitable for yellow fever transmission (details available from the corresponding author on request).

**Fig. 2:**
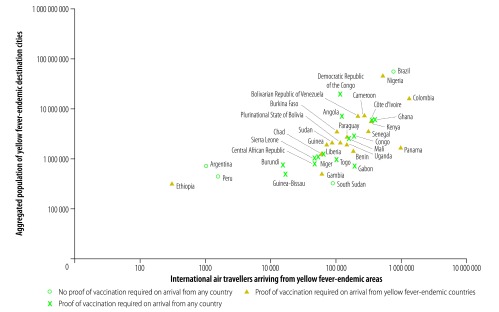
International air travellers arriving from yellow fever-endemic areas and aggregated population of yellow fever-endemic destination cities, by country, 2016

Notes: Both axes have a logarithmic scale. A yellow fever-endemic area was a national or subnational area where the World Health Organization recommended yellow fever vaccination. The symbols indicate the national yellow fever vaccination policy for travellers arriving in the country.

**Fig. 3:**
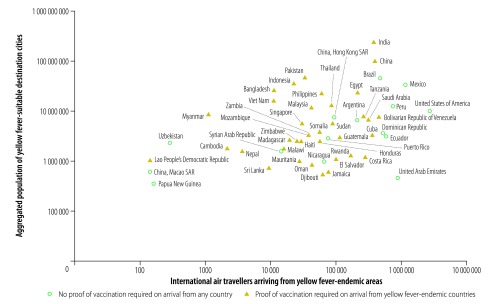
International air travellers arriving from yellow fever-endemic areas and aggregated population of yellow fever-suitable destination cities, by country or territory, 2016

**Fig. 4:**
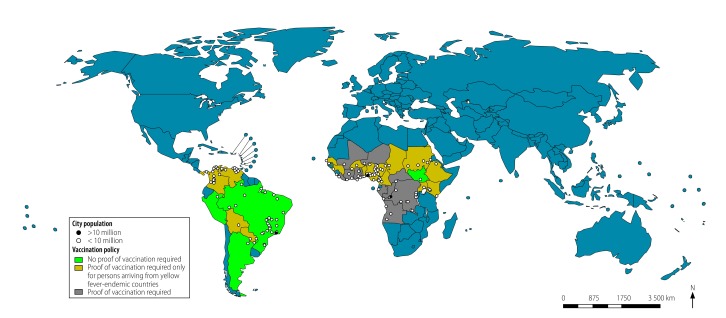
Population of yellow fever-endemic cities, by travel vaccination policy, 2016

Notes: In total, there were 170 yellow fever-endemic cities, represented by circles on the map, in 35 countries. Yellow fever-endemic cities were located in areas where the World Health Organization recommended yellow fever vaccination. In the urban scenario (see main text for details), there was one fewer yellow fever-endemic city than in the base scenario: Tshikapa, Democratic Republic of the Congo (population 0.69 million).

**Fig. 5:**
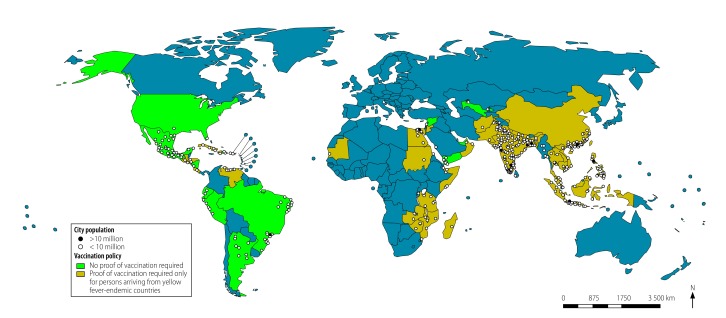
Population of yellow fever-suitable cities, by travel vaccination policy, 2016

Notes: In total, there were 472 yellow fever-suitable cities in 54 countries. A yellow-fever-suitable city was ecologically suitable for yellow fever virus transmission but was not located in a yellow fever-endemic area, which was defined as an area where the World Health Organization recommended yellow fever vaccination. In the urban scenario (see main text for details), there were six fewer yellow fever-suitable cities than in the base scenario: Satna, India (population 0.31 million); Ibb, Yemen (population 0.45 million); Al Hudaydah, Yemen (population 0.57 million); Taiz, Yemen (population 0.69 million); Aden, Yemen (population 0.88 million); and Sana’a, Yemen (population 2.7 million).

Among countries with yellow fever-endemic cities, Brazil, Colombia and Nigeria had the highest traveller numbers from other yellow fever-endemic areas of the world and the largest populations living in yellow fever-endemic cities (Fig. 2). Colombia and Nigeria required proof of yellow fever vaccination from travellers arriving from other yellow fever-endemic countries but not from non-endemic countries. In contrast, Brazil did not require proof of vaccination from travellers arriving from yellow fever-endemic countries. Among countries with yellow fever-suitable cities, Brazil, China, India, Mexico, Peru and the United States had the highest traveller numbers arriving from yellow fever-endemic areas and the largest populations living in yellow fever-suitable cities (Fig. 3). Of these, Brazil, Mexico, Peru and the United States did not require proof of yellow fever vaccination from travellers arriving from yellow fever-endemic areas. Fig. 4 and Table 3 (available at: http://www.who.int/bulletin/volumes/96/5/17-205658) show the resident populations of yellow fever-endemic cities globally according to national yellow fever travel vaccination policy and Fig. 5 and Table 4 (available at: http://www.who.int/bulletin/volumes/96/5/17-205658) show the corresponding populations of yellow fever-suitable cities.

SAR: Special Administrative Region.

Notes: Both axes have a logarithmic scale. A yellow fever-suitable city was ecologically suitable for yellow fever virus transmission but was not located in a yellow fever-endemic area, which was defined as an area where the World Health Organization recommended yellow fever vaccination. The symbols indicate the national yellow fever vaccination policy for travellers arriving in the country.

**Table 3:** Top 50 yellow fever - endemic destination cities of air travellers from areas or cities where yellow fever was endemic, by city population, 2016

Destination city, country or territory,^a^ by rank^b^	Population^c^	Proof of yellow fever vaccination required upon arrival^d^	
		From yellow fever-endemic countries only	From any country
1. Lagos, Nigeria	13 122 829	Yes	No
2., Rio de Janeiro Brazil	12 902 306	No	No
3. Kinshasa, Democratic Republic of the Congo	11 586 914	No	Yes
4. Belo Horizonte, Brazil	5 716 422	No	No
5. Luanda, Angola	5 506 000	No	Yes
6. Abidjan, Côte d'Ivoire	4 859 798	No	Yes
7. Brasília, Brazil	4 155 476	No	No
8. Nairobi, Kenya	3 914 791	Yes	No
9. Medellín, Colombia	3 910 989	Yes	No
10. Porto Alegre, Brazil	3 602 526	No	No
11. Kano, Nigeria	3 587 049	Yes	No
12. Salvador, Brazil	3 582 967	No	No
13. Dakar, Senegal	3 520 215	Yes	No
14. Ibadan, Nigeria	3 160 190	Yes	No
15. Yaoundé, Cameroon	3 065 692	Yes	No
16. Campinas, Brazil	3 047 102	No	No
17. Douala, Cameroon	2 943 318	Yes	No
18. Ouagadougou, Burkina Faso	2 741 128	Yes	No
19. Cali, Colombia	2 645 941	Yes	No
20. Kumasi, Ghana	2 598 789	No	Yes
21. Bamako, Mali	2 515 000	No	Yes
22. Abuja, Nigeria	2 440 242	Yes	No
23. Asunción, Paraguay	2 356 174	Yes	No
24. Port Harcourt, Nigeria	2 343 309	Yes	No
25. Goiânia, Brazil	2 284 828	No	No
26. Accra, Ghana	2 277 298	No	Yes
27. Maracaibo, Venezuela (Bolivarian Republic of)	2 196 435	Yes	No
28. Belém, Brazil	2 181 607	No	No
29. Santa Cruz, Bolivia (Plurinational State of)	2 106 682	Yes	No
30. Manaus, Brazil	2 025 379	No	No
31. Lubumbashi, Democratic Republic of the Congo	2 015 091	No	Yes
32. Mbuji-Mayi, Democratic Republic of the Congo	2 006 641	No	Yes
33. Barranquilla, Colombia	1 991 158	Yes	No
34. Conakry, Guinea	1 936 045	Yes	No
35. Kampala, Uganda	1 935 654	Yes	No
36. Brazzaville, Congo	1 887 625	No	Yes
37. Ciudad de Panama, Panama	1 672 810	Yes	No
38. Grande Vitória, Brazil	1 636 141	No	No
39. Benin City, Nigeria	1 495 763	Yes	No
40. Grande São Luis, Brazil	1 436 781	No	No
41. Huambo, Angola	1 269 211	No	Yes
42. Monrovia, Liberia	1 263 800	No	Yes
43. N'Djaména, Chad	1 260 146	Yes	No
44. Bucaramanga, Colombia	1 215 066	Yes	No
45 Kananga, Democratic Republic of the Congo	1 168 687	No	Yes
46. Onitsha, Nigeria	1 109 287	Yes	No
47. Mombasa, Kenya	1 103 703	Yes	No
48. Cartagena, Colombia	1 092 336	Yes	No
49. Niamey, Niger	1 089 589	No	Yes
50. Kaduna, Nigeria	1 047 815	Yes	No

^a^ All destination countries and territories were yellow fever-endemic areas.

^b^ Cities were ranked according to urban population size.

^c^ We obtained population data from United Nations’ World Urbanization Prospects.^22^

^d^ We did not take into account Brazil’s temporary yellow fever vaccination requirements for incoming passengers from Angola and the Democratic Republic of the Congo during the 2016 outbreak.

Notes: Travel was estimated using our base scenario which considered international travellers arriving from airports within areas where WHO recommended yellow fever vaccination and all airports within 200 km of such areas.Tabulated data reflects cities depicted in Figure 4.

**Table 4:** Top 50 yellow fever suitable destinations, by population, of international air travellers from areas or cities where yellow fever was endemic, by city population, 2016

Destination city, country or territory,^a^ by rank^b^	Population^c^	Proof of yellow fever vaccination required upon arrival^d^		Non-holoendemic country^e^
		From yellow fever-endemic countries only	From any country	
1. New Delhi; India	25 703 168	Yes	No	No
2. São Paulo, Brazil	21 066 245	No	No	Yes
3. Mumbai, India	21 042 538	Yes	No	No
4. Cairo, Egypt	18 771 769	Yes	No	No
5. Dhaka, Bangladesh	17 598 228	Yes	No	No
6. Karachi, Pakistan	16 617 644	Yes	No	No
7. Kolkata, India	14 864 919	Yes	No	No
8. Manila, Philippines	12 946 263	Yes	No	No
9. Guangzhou, China	12 458 130	Yes	No	No
10. Shenzhen, China	10 749 473	Yes	No	No
11. Jakarta, Indonesia	10 323 142	Yes	No	No
12. Bangalore, India	10 087 132	Yes	No	No
13. Lima, Peru	9 897 033	No	No	Yes
14. Chennai, India	9 890 427	Yes	No	No
15. Bangkok, Thailand	9 269 823	Yes	No	No
16. Hyderabad, India	8 943 523	Yes	No	No
17. Lahore, Pakistan	8 741 365	Yes	No	No
18. Dongguan, China	7 434 935	Yes	No	No
19. Ahmadabad, India	7 342 850	Yes	No	No
20. Hong Kong SAR, China	7 313 557	No	No	No
21. Ho Chi Minh City, Viet Nam	7 297 780	Yes	No	No
22. Foshan, China	7 035 945	Yes	No	No
23. Kuala Lumpur, Malaysia	6 836 911	Yes	No	No
24. Miami, United States	5 817 221	No	No	No
25. Pune, India	5 727 530	Yes	No	No
26. Surat, India	5 650 011	Yes	No	No
27. Singapore, Singapore	5 618 866	Yes	No	No
28. Khartoum, Sudan	5 129 358	Yes	No	Yes
29. Dar es Salaam, United Republic of Tanzania	5 115 670	Yes	No	No
30. Guadalajara, Mexico	4 843 241	No	No	No
31. Yangon, Myanmar	4 801 930	Yes	No	No
32. Chittagong, Bangladesh	4 539 393	Yes	No	No
33. Monterrey, Mexico	4 512 572	No	No	No
34. Xiamen, China	4 430 081	Yes	No	No
35. Jiddah, Saudi Arabia	4 075 803	Yes	No	No
36. Shantou, China	3 948 813	Yes	No	No
37. Fortaleza, Brazil	3 880 202	No	No	Yes
38. Recife, Brazil	3 738 526	No	No	Yes
39. Zhongshan, China	3 691 360	Yes	No	No
40. Hà Noi, Viet Nam	3 629 493	Yes	No	No
41. Faisalabad, Pakistan	3 566 952	Yes	No	No
42. Curitiba, Brazil	3 473 681	No	No	Yes
43. Jaipur, India	3 460 701	Yes	No	No
44. Fuzhou, China	3 282 932	Yes	No	No
45. Nanning, China	3 234 379	Yes	No	No
46. Lucknow, India	3 221 817	Yes	No	No
47. Wenzhou, China	3 207 846	Yes	No	No
48. Kanpur, India	3 020 795	Yes	No	No
49. Sana'a', Yemen	2 961 934	No	No	No
50. Santo Domingo, Dominican Republic	2 945 353	No	No	No

SAR: Special Administrative Region.

^a^ Destination cities in these countries and territories were ecologically suitable for yellow fever virus transmission but were not in yellow fever-endemic areas.

^b^ Cities were ranked according to urban population size.

^c^ We obtained population data from United Nations’ World Urbanization Prospects.^22^

^d^ We did not take into account Brazil’s temporary yellow fever vaccination requirements for incoming passengers from Angola and the Democratic Republic of the Congo during the 2016 outbreak.

^e^ Non-holoendemic countries have subnational areas that are at risk of yellow fever transmission as defined by the WHO and CDC Yellow Book. Cities listed in this table are not located within the YF extent of non-holoendemic countries.

Notes: Travel was estimated using our base scenario which considered international travellers arriving from airports within areas where WHO recommended yellow fever vaccination and all airports within 200 km of such areas.Tabulated data reflects cities depicted in Figure 5.

## Discussion

The 2016 yellow fever epidemic in Angola and the associated exportation of cases into urban areas of China exposed shortcomings in existing yellow fever travel vaccination policies and practices. As a holoendemic country, Angola has a policy that requires all international travellers to provide proof of yellow fever vaccination upon arrival. In addition, China has the same requirement for travellers arriving from yellow fever-endemic countries. Yet both lines of defence failed, leading to the first cases of imported yellow fever in Asia. Recent research has confirmed the role played by air travel between Angola and China in increasing the risk of importing the disease.^28^ This event illustrates that urban areas that have never experienced yellow fever transmission, or have not experienced it in modern times, are increasingly susceptible to epidemics. We elected to study the travel conduits that could facilitate the international spread of yellow fever virus into the world’s cities.

First, our analysis revealed that 89% of travellers departing from yellow fever-endemic areas for yellow fever-endemic cities in other countries (both holoendemic and non-holoendemic) in 2016 were required to provide proof of vaccination upon arrival. This high proportion presumably reflects countries’ desire to protect themselves against importation of yellow fever virus. To reduce the risk of importation, and of the consequent potential for domestic transmission and of possible exportation of yellow fever virus, these countries should focus on implementing existing yellow fever travel vaccination policies effectively. However, some travellers may purchase counterfeit international vaccination certificates,^29^ which makes this line of defence potentially fallible. Second, we found that less than 35% of travellers departing yellow fever-endemic areas for cities that appeared suitable for yellow fever transmission, were required to provide proof of vaccination upon arrival. Countries that did not require proof of yellow fever vaccination might have assumed that the historical absence of yellow fever was predictive of its future absence. In other instances, nationally implemented vaccination policies may be obfuscated because only a small geographical area within a country may be ecologically suitable for yellow fever transmission; for example, the 9.5 million United States’ residents who live in five urban areas that appear suitable for yellow fever transmission represent less than 3% of the country’s population. Nonetheless, countries should carefully consider whether the risk of yellow fever virus importation and subsequent domestic transmission warrants a change to existing yellow fever travel vaccination policies or practices. Of note, administering yellow fever vaccine at national ports of entry to individuals who do not hold a record of vaccination will increase immunity among susceptible travellers but will not prevent importation of the virus by travellers who are already infected. Third, we found that less than 25% of travellers who departed from areas of the world where yellow fever was not endemic for yellow fever-endemic cities were required to provide proof of vaccination upon arrival. This reveals a policy gap in protecting international travellers against becoming infected and subsequently exporting the virus. This low proportion may reflect the absence of national incentives because countries with entry requirements for yellow fever vaccination are protecting international travellers and the global community without realizing any domestic benefit.

Although broader use of yellow fever vaccine by international travellers could limit dispersion of the virus and reduce the risk of urban epidemics, its use in non-epidemic settings must be carefully weighed against the risk of vaccine-associated neurological and viscerotropic events. Infants younger than 9 months, adults aged 60 years and older and individuals with thymus disorders and weakened immune systems are at an elevated risk of these potentially life-threatening events.^30^ Furthermore, if international changes in vaccination policy and practice are implemented and enforced, travellers could face difficulties accessing yellow fever vaccine, given current diminished stocks and constrained manufacturing capacity. Even though an estimated 50 million vaccine doses were produced in 2017,^11^ a new yellow fever epidemic in a populated urban centre could readily deplete global emergency vaccine stockpiles.

We made several important assumptions in our analysis. First, we assumed that the risk of yellow fever virus dispersion across all yellow fever-endemic areas of the world was uniform, because we were not attempting to model the spread of the virus out of a particular geographical area that was experiencing epizootic or epidemic activity. Rather, our goal was to describe global pathways via which the yellow fever virus could disseminate to trigger epidemics in the world’s cities, thereby identifying crucial gaps in existing yellow fever travel vaccination policies and practices. Since the potential for international dispersion of the virus out of rural areas presumably differs from that out of urban areas, our urban scenario focused solely on travellers departing airports in or immediately adjacent to cities in yellow fever-endemic areas. However, the recent case of a traveller who acquired a yellow fever virus infection in rural Suriname and then flew to the Netherlands indicates that there is still a risk of yellow fever exportation from rural areas.^4^

Our assumptions about the suitability of cities for yellow fever virus transmission were based on a global ecological model of dengue virus transmission. A recently published modelling analysis of suitability for yellow fever transmission globally predicted a similar pattern to the pattern of dengue suitability we assumed,^31^ especially in urbanized regions, which were the primary focus of our study. However, we may have overestimated the risk of yellow fever transmission in areas where dengue is known to be active but where *Ae. albopictus* rather than *Ae. aegypti* is the dominant vector (e.g. in China, Hong Kong Special Administrative Region). On the other hand, although *Ae. aegypti* is the primary vector for transmission of yellow fever virus, some studies have indicated that *Ae. albopictus* might also be a competent vector in nature.^32^ As our analysis focused on the importation of yellow fever virus into cities and ignored downstream transmission among non-human primates in rural sylvatic cycles, we believe our model of urban dengue suitability closely approximates suitability for yellow fever virus transmission.

Our model of dengue suitability represents an annualized view of potential yellow fever transmission. The model does not account for seasonal variability due to changing climatic conditions.^33^ Furthermore, we did not take into account seasonal patterns in local (i.e. urban–rural) or international travel despite the possibility that interactions between the ecological seasonality of yellow fever transmission and the seasonality of human mobility could influence the risk of yellow fever virus importation. In addition, we did not attempt to quantify variations in the intensity of transmission between tropical and subtropical climates or between industrialized and developing areas of the world. For example, because of differences in climate and the built environment,^34^ some cities in the southern United States have experienced sporadic transmission of dengue, chikungunya and Zika viruses, whereas cities in Latin America have experienced sustained and intense transmission of the same pathogens. Moreover, we did not attempt to estimate how the underlying level of population immunity influences the potential for epidemics. Although we presumed that populations in yellow fever-suitable cities would have negligible immunity to the yellow fever virus, we made no assumptions about immunity in yellow fever-endemic cities, because high-resolution data on yellow fever vaccination and natural infection were lacking. Lastly, we did not take into account Brazil’s temporary yellow fever vaccination requirements for travellers who came from Angola and the Democratic Republic of the Congo during the 2017 yellow fever outbreak and therefore categorized Brazil as not requiring proof of vaccination upon arrival from yellow fever-endemic countries.

With more than 3 billion domestic and international passengers now boarding commercial flights each year, humans have become the primary agents for the global spread of mosquito-borne viruses such as dengue, chikungunya, Zika and yellow fever. Our findings on yellow fever virus transmission provide countries with insights into contemporary vulnerabilities to international spread of the virus. Our goal was to help countries ensure that their policies and interventions to prevent, or to protect against, the international spread of yellow fever virus are commensurate with existing risks and avoid unnecessary interference with international traffic and trade, as per International Health Regulations (2005).^35^ At a time when global yellow fever vaccine supplies are diminished, an epidemic in a densely populated city could have substantial health and economic consequences. Hence, the global community needs to carefully re-examine existing yellow fever travel vaccination policies and practices to prevent urban epidemics.
